# Method for estimating disease risk from microbiome data using structural equation modeling

**DOI:** 10.3389/fmicb.2023.1035002

**Published:** 2023-01-26

**Authors:** Hidetaka Tokuno, Tatsuya Itoga, Jumpei Kasuga, Kana Okuma, Kazumi Hasuko, Hiroaki Masuyama, Yoshimi Benno

**Affiliations:** ^1^Symbiosis Solutions Inc., Tokyo, Japan; ^2^Benno Institute for Gut Microflora, Saitama, Japan

**Keywords:** microbiota, human intestinal microbiome, atopic dermatitis, amplicon sequence variants, structural equation modeling, latent variables

## Abstract

The relationship between the human gut microbiota and disease is of increasing scientific interest. Previous investigations have focused on the differences in intestinal bacterial abundance between control and affected groups to identify disease biomarkers. However, different types of intestinal bacteria may have interacting effects and thus be considered biomarker complexes for disease. To investigate this, we aimed to identify a new kind of biomarker for atopic dermatitis using structural equation modeling (SEM). The biomarkers identified were latent variables, which are complex and derived from the abundance data for bacterial marker candidates. Groups of females and males classified as healthy participants [normal control (NC) (female: 321 participants, male: 99 participants)], and patients afflicted with atopic dermatitis only [AS (female: 45 participants, male: 13 participants)], with atopic dermatitis and other diseases [AM (female: 75 participants, male: 34 participants)], and with other diseases but without atopic dermatitis [OD (female: 1,669 participants, male: 866 participants)] were used in this investigation. The candidate bacterial markers were identified by comparing the intestinal microbial community compositions between the NC and AS groups. In females, two latent variables (lv) were identified; for lv1, the associated components (bacterial genera) were *Alistipes*, *Butyricimonas*, and *Coprobacter*, while for lv2, the associated components were *Agathobacter*, *Fusicatenibacter*, and *Streptococcus*. There was a significant difference in the lv2 scores between the groups with atopic dermatitis (AS, AM) and those without (NC, OD), and the genera identified for lv2 are associated with the suppression of inflammatory responses in the body. A logistic regression model to estimate the probability of atopic dermatitis morbidity with lv2 as an explanatory variable had an area under the curve (AUC) score of 0.66 when assessed using receiver operating characteristic (ROC) analysis, and this was higher than that using other logistic regression models. The results indicate that the latent variables, especially lv2, could represent the effects of atopic dermatitis on the intestinal microbiome in females. The latent variables in the SEM could thus be utilized as a new type of biomarker. The advantages identified for the SEM are as follows: (1) it enables the extraction of more sophisticated information when compared with models focused on individual bacteria and (2) it can improve the accuracy of the latent variables used as biomarkers, as the SEM can be expanded.

## 1. Introduction

Human gut microbiome research has been notably enhanced by the recent advances in bacterial isolation (Moore and Holdeman, 1974), culture techniques (Hayashi et al., 2002), and phylogenetic classification (Rajilić-Stojanović and de Vos, 2014). In recent years, large-scale metagenomic analyses have become available, which have enabled the elucidation of the gut microbiome diversity among the population (Cénit et al., 2014) and its associations with disease [e.g., obesity (Turnbaugh et al., 2009), type 2 diabetes (Vijay and Valdes, 2022), atherosclerosis (Karlsson et al., 2012; Yoshida et al., 2018), Crohn’s disease (Sokol et al., 2008), allergic diseases (Lunjani et al., 2018), autoimmune diabetes (Shimokawa et al., 2020), colorectal cancer (Ahn et al., 2013), chronic obstructive pulmonary disease (Bowerman et al., 2020), and multiple sclerosis disease (Miyake et al., 2015)]. There has been significant interest in developing risk assessment methods based on gut microbiome data (Li et al., 2017; Xu et al., 2019; Chu et al., 2021; Loftus et al., 2021; Shen et al., 2021; Yao et al., 2021). Attempts have also being made to identify gut bacteria as biomarkers of disease (Yu et al., 2017; Loftus et al., 2021; Yao et al., 2021; Lu et al., 2022). For example, Yao et al. (2021) proposed the use of *Fusobacterium* as an indicator for colorectal cancer (detection) based on the results of linear discriminant analysis and effect size, while Lu et al. (2022) identified 15 genera, including *Lactobacillus*, *Bifidobacterium*, and *Akkermansia*, using a random forest classification model as biomarkers (targeted biomarkers) for cognitive impairment. Most previous studies have focused on individual intestinal bacteria. However, there are over 1,000 species of intestinal bacteria in the human gut (Rajilić-Stojanović and de Vos, 2014; Yang et al., 2020), and their ecosystem is diverse and complex. Consequently, the idea that only one or several specific genera or species of gut bacteria are associated with any single disease seems illogical. However, the previous models that have been used to assess disease risk either use a single bacterium as a biomarker, such as Yao et al. (2021), or multiple bacteria independently as variables, such as Lu et al. (2022), and they do not consider the interactions among bacteria. Given the complexity of the intestinal microbiome, it is difficult to obtain a complete picture of the association between the intestinal microbiome and disease, or to assess the risk of disease from the intestinal microbiome simply by analyzing the association between individual intestinal bacteria and disease.

We therefore propose the use of structural equation modeling (SEM; Tarka, 2018) as a method to solve this problem. In SEM, both factor analysis (Howard, 2016) and path analysis (Streiner, 2005) are performed. For factor analysis, latent variables, which are based on the common results for several observed variables (in the case of this study, variables representing the abundance of each genus) are determined using variance results and summarized in one factor (Dragan and Topolšek, 2014) for use in further analysis. This is thought to reflect to some extent, the interactions of multiple observed variables. Since the value of this latent variable can be obtained for each participant (DiStefano et al., 2009), it is possible to calculate and use features that aggregate multiple observed variables with common characteristics for each participant. In addition, in SEM, latent variables can be freely set based on prior hypotheses (De Carvalho and Chima, 2014), and the observed variables used in their synthesis. Thus, the interpretation of the composite (latent) variables is easier than in principal component analysis (Wold et al., 1987), where all observed variables are aggregated to create a composite variable. In addition, observed variables can be added at will; for example, if a latent variable is estimated to be related to anti-inflammatory effects in the body, it can be improved by adding a new observed variable that is related to anti-inflammatory effects. This study has utilized a large Japanese intestinal microbial community composition database, which is obtained with 16S rRNA amplicon sequencing analysis. The database consists of 14,693 samples (4,907 males and 9,786 females aged ∼20–79 years), which were collected by the Benno Laboratory of RIKEN (Wako, Saitama, Japan) and analyzed by the Japan Agricultural Frontier Development Organization. In addition to the intestinal microflora analysis data, information on lifestyle habits such as diet and disease status were obtained from self-reported questionnaires. The database contains information on approximately 940 diseases according to the International Statistical Classification of Diseases and Related Health Problems (International Statistical Classification of Diseases and Related Health Problems, Tenth: ICD-10). Using this database, models can be constructed based on sex and disease, using detailed information such as age, sex, and disease status. In this study, we have proposed a new method to analyze the relationship between human intestinal microbiota and disease and estimate the degree of likelihood of contracting a particular disease (disease risk) using intestinal microbiota analysis data. The effectiveness (application to clinical practice) of this method was assessed using atopic dermatitis as a case study, as numerous reports have shown a relationship between this disease and intestinal bacteria (Park et al., 2020; Ye et al., 2021).

## 2. Materials and methods

### 2.1. Ethical considerations

This investigation utilized stool samples and questionnaire responses that had been collected through the ONAKA Care Project (20 September 2011–31 March 2021) conducted by the Benno Special Laboratory (Wako City, Saitama Prefecture, Japan) at the Institute of Physical and Chemical Research (RIKEN), Japan. This project was approved by the Research Ethics Committee of RIKEN (approval number: Wako 3 27-22), and consent to research participation was obtained from all stool sample providers and questionnaire respondents.

### 2.2. Sample collection

**FIGURE 1:**
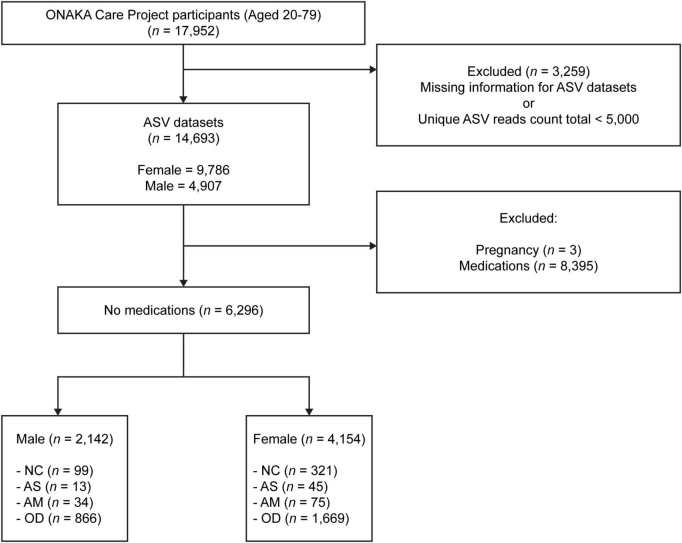
Flowchart showing the target groups analyzed in this study. There were a total 17,952 samples, each from a different Japanese participant aged 20–79 years, which were collected through the ONAKA Care Project. The amplicon sequence variant (ASV) data was obtained using 14,693 of the 17,952 samples. NC, normal controls; AS, patients with atopic dermatitis without other diseases; AM, patients with atopic dermatitis with other diseases; OD, patients with other diseases but without atopic dermatitis. Please refer to the (Section “2. Materials and methods”) subsections (Section “2.7.1. Normal control group”, “2.7.2. Patients with atopic dermatitis and no other diseases”, “2.7.3. Patients with atopic dermatitis and other diseases”, and “2.7.4. Patients without atopic dermatitis but with other diseases”) for details on the criteria used to define each group.

Stool samples from 17,952 (12,029 females and 5,923 males) different Japanese people aged 20–79 years were obtained for the ONAKA Care Project by the Benno Special Laboratory (Wako City, Saitama Prefecture, Japan) at the Institute of Physical and Chemical Research (RIKEN), Japan (Figure 1). Project participants were recruited using Japanese newspaper advertisements and announcements at public lectures. The stool samples were collected by the participants themselves using a stool collection brush type kit (TechnoSuruga Laboratory Co., Ltd., Shizuoka, Japan) in accordance with the manufacturer’s instructions. The samples were mailed with no temperature control after being suspended in a guanidine thiocyanate solution [100 mM Tris–HCL (pH 8.0), 40 mM Tris-EDTA (pH 9.0), 4 M guanidine thiocyanate, 0.001% bromothymol blue].

### 2.3. Questionnaire survey

A questionnaire survey was conducted with the 17,952 participants who provided stool samples to gather information including the participants’ sex, age, body mass index (BMI), defecation frequency, lifestyle (e.g., drinking, smoking, exercise frequency), morbidity, sleep status, the Center for Epidemiologic Studies Depression scale (CES-D), presence or absence of *Helicobacter pylori* treatment, hospitalization or surgery experience, status for prescription or over-the-counter medications, and for females only, their menstrual status and whether they were pregnant or breastfeeding. Disease morbidity was divided into 11 categories (atopic dermatitis, bone and/or joint disease, bronchial asthma, diabetes, dyslipidemia, gastrointestinal disease, heart disease, hyperpiesia, kidney disease, liver disease, lower back and/or joint pain) and identified for each participant, including whether or not they were currently being treated. Free-form responses were also be collected for diseases that did not fit in the above 11 categories. Prescription or over-the-counter medication use was divided into 15 categories (gastric/duodenal ulcer/reflux esophagitis, hypertension, hyperlipidemia, diabetes, hypnotics, painkillers/antipyretics, allergy, angina treatment, laxative/constipation, osteoporosis, rheumatism, corticosteroids, antibiotics, cold drugs, and antithrombotic drugs). Free-form responses were also collected for medicines other than those listed in the 15 categories.

### 2.4. DNA extraction

For DNA extraction, glass beads (400 mg) were added to 600 μL of Tris-EDTA saturated phenol/chloroform solution (phenol:chloroform = 1:1) and combined with 100 μL of 10% SDS solution and the guanidine thiocyanate solution, in which there was approximately 4 mg of a stool sample suspended. This mixed solution was homogenized (MagNA Lyser; Roche Molecular Diagnostic, Mannheim, Germany) at 7,000 rpm for 20 s, and then allowed to stand at 70°C for 10 min. This homogenization and standing procedure were conducted twice, and afterward, the sample was cooled in a water bath at 15°C. The sample was then centrifuged at 10,621 × *g* for 5 min at 25–28°C, after which the supernatant was separated. Then, 700 μL of cold isopropyl alcohol and 70 μL of 3 M sodium acetate solution were added to the separated supernatant and mixed by inversion. The mixed solution was centrifuged at 20,800 × *g* for 5 min at 25–28°C, after which the supernatant was removed. The precipitated DNA pellets were washed twice with a 70% ethanol solution and dried using an evaporator (Tokyo Rikakikai Co., Ltd., Tokyo, Japan). The dried DNA pellets were dissolved in 200 μL Tris-EDTA buffer and stored at −80°C.

### 2.5. DNA sequencing

Deoxyribonucleic acid extracted from the stool samples (12.5 ng) was used for DNA sequencing. The variable regions V1–V3 of the 16S rRNA were the sequencing targets, and the PCR amplicons were generated using the 35F primer (5′-TCGTCGGCAGCGTCAGATGTGTATAAGAGACAGCCTGGCAC AGGATGAACG-3′; Hayashi et al., 2004) and 520R primer (5′-GTCTCGTGGGCTCGGAGATGTGTATAAGACAGACCGCGGCT TG-3′). The PCR program used 20 cycles (98°C, 10 s; 55°C, 30 s; 72°C, 60 s) with Q5 High-Fidelity DNA Polymerase (New England Biolabs, Ipswich, MA, USA). Agarose gel electrophoresis confirmed the amplification of the target length fragment, and the PCR-amplified fragment was purified using AMPure XP Reagent (Beckman Coulter, Brea, CA, USA). Index PCR was conducted with the purified PCR-amplified fragments using the Nextera XT Index Kit v2 primers (Illumina, San Diego, CA, USA) for eight cycles (98°C, 10 s; 55°C, 30 s; 72°C, 60 s). The concentration of the index PCR product was measured using the QuantiFluor dsDNA System (Promega, Madison, WI, USA), and the index PCR product was diluted to 4 nM. For each sample, 5 μL was collected and used to make one library and then denatured with 0.2 N aqueous sodium hydroxide solution to adjust the final concentration to 8 p.m. Then, 8 p.m of the denatured PhiX was added to the solution to generate a final concentration of 25–40% (v/v). This prepared solution was treated with a MiSeq system (Illumina, San Diego, CA, USA) that used Reagent Kit v3, and DNA sequencing was conducted using 300 cycles, twice with the MiSeq following the manufacturer’s settings.

### 2.6. 16S rRNA data analysis

A fastq file was created from the base call (bcl) file output of the MiSeq DNA sequencing using the bcl2fastq software (version 2.20.0.422; Illumina). The fastq file was processed using the clsplitseq software (version 0.2.2019.05.10),^1^ and the primer sequences were removed to create the demultiplexed fastq file. The quality score for clsplitseq was set to 20. The generated overlapping and paired-end fastq file was processed using the DADA2 package (version 1.16; Callahan et al., 2017) in R (R Foundation for Statistical Computing, Vienna, Austria) to create amplicon sequence variants (ASVs; Callahan et al., 2017). The DADA2 package was run according to the DADA2 pipeline tutorial version 1.16^2^ and DADA2 workflow for Big Data version 1.4 or later.^3^ Here, the arguments of the filterAndTrim function included truncLen = c (0,0), maxN = 0, maxEE = 5, truncQ = 4, rm.phix = TRUE, compress = TRUE, multithread = TRUE, verbose = TRUE, and minLen = 50. A sample was extracted in which the total number of reads for each unique ASV sequence created was ≥ 5,000, and coverage-based rarefying (Chao and Jost, 2012) was conducted using the vegan package (version 2.5.7; Oksanen et al., 2020) based on the number of reads for each unique ASV sequence. Silva version 132 was used to identify the genus name for each uniquely created ASV sequence. Finally, ASV microbial community composition data were obtained for 14,693 of the 17,952 participants, as the data for 3,259 participants were excluded due to low quality read counts or missing bcl files (Figure 1).

### 2.7. Setting the analysis target groups

The following analysis groups were defined for each sex, using the available data for the 14,693 participants from which the ASV data were obtained, using a self-reported questionnaire. The number of participants (Figure 1), distribution of age, BMI, CES-D (Table 1), and disease morbidity (Table 2) for each of the groups has been presented in the respective figures and tables.

**TABLE 1:** Age, Center for Epidemiologic Studies Depression scale, and body mass index distributions for the analyzed groups.

Metadata^1^	Statistic^2^	Male (*n* = 2,142)				Female (*n* = 4,154)			
		NC^3^ (*n* = 99)	AS^4^ (*n* = 13)	AM^5^ (*n* = 34)	OD^6^ (*n* = 866)	NC^3^ (*n* = 321)	AS^4^ (*n* = 45)	AM^5^ (*n* = 75)	OD^6^ (*n* = 1,669)
Age	Min	20	26	21	20	20	21	20	20
	1st Qu	32	35	36	41	39	34	34	42
	Median	41	37	42.5	51	50	39	40	50
	Mean	41.39	39.77	42.53	51.11	48.96	40.93	41.11	49.82
	3rd Qu	48	43	48.75	61.75	60	49	45.5	59
	Max.	79	64	75	79	78	70	72	79
	NAs	–	–	–	–	–	–	–	–
CES-D	Min	0	1	4	0	0	0	0	0
	1st Qu	3.5	2	8.25	5	3	4	8.5	6
	Median	7	6	13.5	10	7	5	16	11
	Mean	6.919	6.154	13.68	11.55	6.782	6.533	16.44	12.81
	3rd Qu	10	9	18	16	10	11	22	18
	Max	15	15	39	48	15	15	43	58
	NA’s	–	–	–	–	–	–	–	–
BMI	Min	18.51	18.61	17.3	13.63	18.51	18.54	15.24	11.43
	1st Qu	20.48	19.96	19.79	21.11	19.63	19.11	18.37	18.36
	Median	21.45	21.26	21.88	23.01	20.69	20.61	19.98	20.44
	Mean	21.71	21.25	22.32	23.27	20.92	20.61	20.27	21.08
	3rd Qu	23.09	21.97	24.66	25.4	21.91	21.62	21.46	23.02
	Max	24.69	23.94	32.04	46.17	24.92	24.53	28.48	44
	NA’s	–	–	–	–	–	–	–	1

^1^CES-D, the Center for Epidemiologic Studies Depression scale; BMI, body mass index.

^2^Min., 1st Qu, Median, Mean, 3rd Qu, and Max. Represent the minimum value, first quartile, median, mean, third quartile, maximum, respectively. NA represents the number of patients with missing values for each item (age, CES-D, or BMI).

^3^Normal controls.

^4^Patients with atopic dermatitis without another disease.

^5^Patients with atopic dermatitis with other diseases.

^6^Patients with other diseases but without atopic dermatitis.

**TABLE 2:** Disease morbidity in the target populations analyzed.

Disease name	Count							
	Male (*n* = 2,142)				Female (*n* = 4,154)			
	NC^1^ (*n* = 99)	AS^2^ (*n* = 13)	AM^3^ (*n* = 34)	OD^4^ (*n* = 866)	NC^1^ (*n* = 321)	AS^2^ (*n* = 45)	AM^3^ (*n* = 75)	OD^4^ (*n* = 1,669)
Allergic rhinitis	0	0	0	3	0	0	0	12
Anemia	0	0	0	1	0	0	2	25
Angina pectoris	0	0	0	1	0	0	5	2
Apnea syndrome	0	0	0	11	0	0	0	2
Arrhythmia	0	0	0	9	0	0	0	10
Asthma	0	0	2	13	0	0	0	22
Atopic dermatitis	0	13	34	0	0	45	75	0
Autoimmune disease	0	0	0	1	0	0	1	8
BMI < 18.5	0	0	0	56	0	0	0	341
BMI ≥ 25	0	0	0	202	0	0	0	161
Bone joint disease	0	0	0	17	0	0	3	72
Breast cancer	0	0	0	0	0	0	1	17
Cataract	0	0	0	5	0	0	0	7
Cerebrovascular disease	0	0	0	1	0	0	0	4
CES-D ≥ 16	0	0	0	185	0	0	0	393
Colorectal cancer	0	0	0	4	0	0	0	9
Colorectal cancer and/or polyp treatment	0	0	5	162	0	0	3	200
Colorectal polyp	0	0	0	4	0	0	0	6
Constipation	0	0	0	0	0	0	0	0
Depression	0	0	0	1	0	0	0	5
Diabetes	0	0	1	24	0	0	0	17
Dyslipidemia	0	0	1	51	0	0	5	99
Gastritis	0	0	0	0	0	0	0	1
Gastrointestinal disease	0	0	3	25	0	0	1	74
Glaucoma	0	0	0	22	0		0	30
Gout	0	0	1	12	0	0	0	0
Graves Basedow disease	0	0	0	0	0	0	0	5
Hashimoto disease	0	0	0	0	0	0	2	16
Headache	0	0	0	0	0	0	0	3
Heart disease	0	0	0	19	0	0	1	26
Hepatitis	0	0	0	2	0	0	0	10
Hyperpiesia	0	0	2	47	0	0	0	47
Irritable bowel syndrome	0	0	0	3	0	0	1	4
Kidney disease	0	0	0	10	0	0	1	16
Knee osteoarthritis	0	0	0	3	0	0	0	5
Liver disease	0	0	1	8	0	0	0	23
Low back joint pain	0	0	1	89	0	0	16	196
Menopause	0	0	0	0	0	0	0	6
Menstrual disorder	0	0	0	0	0	0	1	3
Myocardial infarction	0	0	0	2	0	0	0	0
Nervousness	0	0	0	1	0	0	0	4
Osteoarthrosis	0	0	0	0	0	0	0	6
Osteoporosis	0	0	0	0	0	0	0	8
Others	0	0	21	42	0	0	39	125
Pollinosis	0	0	0	8	0	0	2	22
Prostate	0	0	0	9	0	0	0	0
Prostatic cancer	0	0	0	5	0	0	0	0
Reflux esophagitis	0	0	0	3	0	0	0	15
Rheumatoid arthritis	0	0	0	0	0	0	0	6
Rhinitis	0	0	0	5	0	0	1	13
Sjogren syndrome	0	0	0	1	0	0	0	5
Sleep disorder	0	0	0	0	0	0	0	1
Thyroid abnormalities	0	0	0	0	0	0	2	42
Ulcerative colitis	0	0	1	5	0	0	0	7
Uterus disease	0	0	0	0	0	0	0	40

^1^Number of normal controls.

^2^Number of patients with atopic dermatitis without other diseases.

^3^Number of patients with atopic dermatitis with other diseases.

^4^Number of patients with other diseases but without atopic dermatitis.

#### 2.7.1. Normal control group

The normal control (NC) group consisted of participants who satisfied conditions (NC1) to (NC13), which were as follows: (NC1) not afflicted by any disease; (NC2) no prescription or over-the-counter drugs taken; (NC3) BMI value ≥ 18.5 and < 25; (NC4) CES-D value < 16; (NC5) no experience of hospitalization or surgery; (NC6) no experience of taking drugs for *Helicobacter* infection at the time of stool collection; (NC7) no experience of medical examinations regarding insomnia at the time of stool collection; (NC8) defecation frequency of 1–2 times a day, or 4–6 times a week; (NC9) frequency of alcohol consumption of ≤ 5 days a week, the amount of which equates to ≤ 180 mL of Japanese sake, or no alcohol consumption; (NC10) no history of smoking; (NC11) regular menstrual cycle, or menopausal, but females aged < 40 and had already undergone menopause were not included in the NC group (conditions for females only); (NC12) not menstruating at the time of stool collection (conditions for females only); (NC13) not pregnant or breastfeeding (conditions for females only).

#### 2.7.2. Patients with atopic dermatitis and no other diseases

Patients with atopic dermatitis and no other diseases (henceforth referred to as AS) were those who satisfied conditions (AS1) to (AS4), which were as follows: (AS1) afflicted with atopic dermatitis; (AS2) not afflicted by any other disease (BMI value < 18.5 or ≥ 25, CES-D of ≥ 16, and those who had previously underwent colorectal cancer/colorectal polyp surgery were considered to have had another disease); (AS3) no prescription or over-the-counter drugs taken; (AS4) not pregnant or breastfeeding (conditions for females only).

#### 2.7.3. Patients with atopic dermatitis and other diseases

Patients with atopic dermatitis and other diseases (henceforth referred to as AM) were those who satisfied conditions (AM1) to (AM4), which were as follows: (AM1) afflicted with atopic dermatitis; (AM2) afflicted by a disease other than atopic dermatitis (BMI value < 18.5 or ≥ 25, CES-D of ≥ 16, and those who previously underwent colorectal cancer/colorectal polyp surgery were considered to have other diseases); (AM3) no prescription or over-the-counter drugs taken; (AM4) not pregnant or breastfeeding (conditions for females only).

#### 2.7.4. Patients without atopic dermatitis but with other diseases

Patients without atopic dermatitis but with other diseases (henceforth referred to as OD) were those who satisfied conditions (OD1) to (OD4), which were as follows: (OD1) not afflicted with atopic dermatitis; (OD2) afflicted by a disease other than atopic dermatitis (BMI value < 18.5 or ≥ 25, CES-D of ≥ 16; and those who previously underwent colorectal cancer/colorectal polyp surgery were considered to have other diseases); (OD3) no prescription or over-the-counter drugs taken; (OD4) not pregnant or breastfeeding (conditions for females only).

### 2.8. CLR transformation of the intestinal microbial community composition data

The microbial community composition data derived from the ASVs were converted using a centered log-ratio (CLR) transformation. The CLR transformation of the intestinal microbial community composition data was conducted using the aldex.clr function in the ALDEx2 package (version 1.24.0; Fernandes et al., 2013) in R (R Foundation for Statistical Computing). At this time, mc.samples = 128 and demon = “all” were set as the arguments of the function. The CLR-transformed data were used in the analyses described in subsections (Sections “2. 9. Identification of genera with expression differences in the intestinal microbiomes of the healthy and atopic dermatitis-afflicted individuals,” “2.10. SEM,” “2.11. Estimation of the latent variable score,” and “2.12. Construction of an atopic dermatitis morbidity probability estimation model”). The CLR transformation was performed with set.seed (1234).

### 2.9. Identification of genera with expression differences in the intestinal microbiomes of the healthy and atopic dermatitis-afflicted individuals

Genera that reflected the differences in the intestinal microbiomes of the healthy and atopic dermatitis-afflicted individuals were identified for the male and female groups. The search was conducted between healthy participants (henceforth referred to as NC) and AS to rule out the effects of diseases other than atopic dermatitis. The effect size between the NC and AS was calculated using the aldex.effect function in the ALDEx2 package. In the ALDEx2 package, Monte Carlo sampling was used in the calculation process (Fernandes et al., 2013), and consequently, it was affected by the pseudo-random simulation of values (Supplementary Figure 1). Therefore, the effect size for both males and females was calculated 500 times and arranged in descending order based on the absolute value of the effect size. In each 500-time calculation, different random numbers were used. In this manner, we obtained 500 lists of the top 20 genera for both males and females and identified the genera that intersected across all lists. We then searched for genera in which the absolute value of a relatively large effect size was calculated in a stable manner even under the influence of random numbers. Calculations of effect sizes were performed with set.seed (1,234).

### 2.10. SEM

We constructed SEM using the CLR-transformed intestinal microbial community composition data from all participants (male or female) in NC and AS groups and the atopic dermatitis morbidity data. The SEM used the cfa function of the lavaan package (version 0.6-9; Rosseel, 2012). At this time, std.lv = TRUE, std.ov = TRUE, and check.gradient = TRUE were set as the function arguments. Additionally, the observed variable that represented atopic morbidity in the structural equation was set as a categorical variable, and the estimation method was set as diagonally weighted least squares. To construct the SEM, we assumed that there were two latent variables (one with a positive effect on atopic dermatitis and the other with a negative effect) that explained the binary categorical variables representing atopic dermatitis morbidity. Indicators were assigned to the latent variables with reference to the result of the effect size calculation described in subsection (Section“2.9. Identification of genera with expression differences in the intestinal microbiomes of the healthy and atopic dermatitis-afflicted individuals”). We used this SEM as a starting point from which to make modifications. The indicators were deleted until no negative components appeared in the variance-covariance matrix calculated in the process. Subsequently, the indicators were deleted so that the *p*-value of each parameter, whose null hypothesis was that the estimated parameters equaled zero, was < 0.05; for the final model, we adopted those with values close to one for their goodness-of-fit index (GFI) and adjusted GFI (AGFI), values close to 0 for the root mean square error of approximation (RMSEA), and the maximum absolute value for the path coefficient from the latent variable to the variable representing disease morbidity. The estimation of each standardized parameter for the SEM was conducted using the standardizedSolution function in the lavaan package. The calculation of each GFI for the SEM was conducted using the fitmeasures function of the lavaan package. The calculations for each latent variable value in the structural equation model were conducted for participants belonging to each of the NC, AS, AM, and OD groups using the lavPredict function in the lavaan package. Here, method = “Bartlett” was set for the argument of the function. The usage of the lavaan package was based on Rosseel (2012), Toyoda (2014), and the lavaan package manual implemented in the package. The latent variable values were compared between groups using the Wilcoxon–Mann–Whitney rank sum test with the ggsignif package (version 0.6.3; Ahlmann-Eltze and Patil, 2021).

### 2.11. Estimation of the latent variable score

A new SEM was constructed by extracting the measurement equation portion of the previously constructed SEM. Each parameter in this model was set to the same value as for the original. The extraction of the measurement equation part of the first SEM was performed as follows:

1. Let *F*_*i*_ (*i* = 1, 2) be a latent variable in first SEM, *x*_*ij*_ [j = 1, 2, …, max(*m*, *n*)] be an indicator of *F*_*i*_, *a*_*ij*_ be a factor loading of *x*_*ij*_ calculated in first SEM, *c* be a covariance between *F*_1_ and *F*_2_ calculated in first SEM, and *v*_*ij*_ be a residual variance of *x*_*ij*_ calculated in first SEM. If *F*_1_ = ∼ *x*_11_ + *x*_12_ + … + *x*_1*n*_, *F*_2_ = ∼ *x*_21_ + *x*_22_ + … + *x*_2*m*_, *y* ∼ F_1_ + F_2_, *F*_1_ ∼∼ *F*_2_ are structural equations of first SEM inputted to the cfa function,
2. Set the structural equation of the new SEM for the cfa function as follows: *F*_1_ = ∼ *a*_11_*x_11_ + *a*_12_*x_12_ + … + *a*_1n_*x_1*n*_, *F*_2_ = ∼ *a*_21_*x_21_ + *a*_22_*x_22_ + … + *a*_2m_*x_2*m*_, *F*_1_ ∼∼ c*F_2_, *x*_*ij*_ ∼∼ *v*_ij_*x_*ij*_.

The SEM used the CLR-transformed intestinal microbial community composition data for all females in the NC and AS groups based on the new SEM and the cfa function. At this time, the std.lv = TRUE, std.ov = TRUE, and check.gradient = TRUE were set as the arguments, and the estimation method was diagonally weighted least squares. Based on the result, the estimated values for each latent variable of the participants belonging to the NC, AS, AM, and OD groups were obtained using the lavPredict function. Here, method = “Bartlett” was set as the argument for the function. The estimations for the latent variable values were compared between groups using the Wilcoxon–Mann–Whitney rank sum test with the ggsignif package.

### 2.12. Construction of an atopic dermatitis morbidity probability estimation model

The merged populations of the NC, AS, AM, and OD groups were randomly divided in a stratified manner with 80% as the training population and 20% as the verification population. The sample_frac and group_by functions of the dplyr package (version 1.0.7; Wickham et al., 2021) were used for the divisions. The number of atopic dermatitis and non-atopic dermatitis patients in the training population was balanced using the synthetic minority over-sampling technique (Chawla et al., 2002) with the smote function of the performanceEstimation (version 1.1.0; Torgo, 2014). A multivariate logistic regression model was used as the model for estimating the atopic dermatitis morbidity probability. The latent variable values of the training populations were used to determine the parameters during model construction. For the prediction models, the estimated latent variable values of the verification population were used. The glm function was used to construct the multivariate logistic regression model and predict the function to fit the verification population to the model. To investigate the accuracy of the models, ROC analysis was conducted using the roc function in the pROC package (version 1.18.0; Robin et al., 2011). The inputs to the function were atopic dermatitis morbidity probability, which was estimated by the models, and the actual atopic dermatitis morbidity for each participant. The ggroc function of the pROC package was used to draw the ROC curves and calculate the area under the curve (AUC) scores. The atopic dermatitis morbidity probability was compared between groups using the Wilcoxon–Mann–Whitney rank sum test in the ggsignif package.

### 2.13. R software versions

The R software used in subsection (2.6. “16S rRNA data analysis”) was R version 4.0.3 (R Foundation for Statistical Computing, R Core Team, 2020). The statistical or data analyses described in subsections (Sections “2.8. CLR transformation of the intestinal microbial community composition data,” “2.9. Identification of genera with expression differences in the intestinal microbiomes of the healthy and atopic dermatitis-afflicted individuals,” “2.10. SEM,” “2.11. Estimation of the latent variable score,” and “2.12. Construction of an atopic dermatitis morbidity probability estimation model”) were performed with R version 4.1.0 (R Foundation for Statistical Computing, R Core Team, 2021).

## 3. Results

### 3.1. Expression differences of the genera in the intestinal microbiome

**FIGURE 2:**
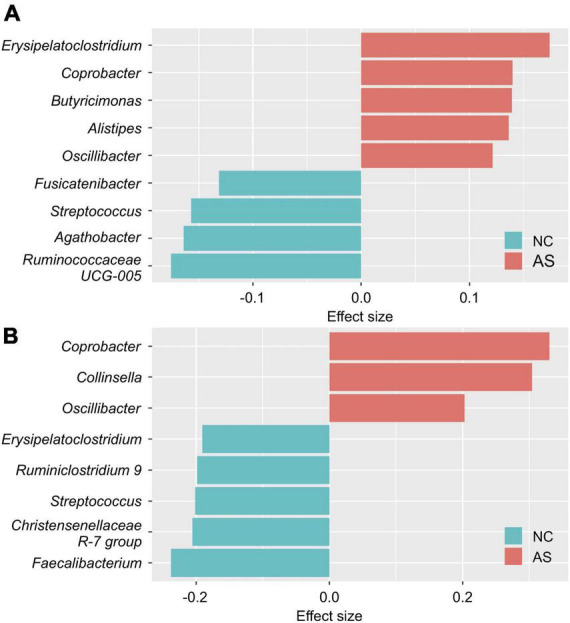
Differences in the intestinal microbiome genera of healthy and atopic dermatitis-afflicted individuals. The effect size between the normal control group (NC) and patients with atopic dermatitis without other diseases (AS) was calculated separately for **(A)** females and **(B)** males using the aldex.effect function in the ALDEx2 package (version 1.24.0; Fernandes et al., 2013). The effect size was calculated 500 times and arranged in descending order based on the absolute value of the effect size. In this manner, we obtained 500 lists of the top 20 genera, and identified the genera intersecting all lists (nine genera for the females and eight for the males). The effect size value is the average value of the 500 calculations.

In females, *Erysipelatoclostridium*, *Coprobacter*, *Butyricimonas*, *Alistipes*, and *Oscillibacter* were found to have an effect on AS; and *Ruminococcaceae UCG-005*, *Agathobacter, Streptococcus*, and *Fusicatenibacter* were found to have an effect on NC (Figure 2A). While in males, *Coprobacter, Collinsella*, and *Oscillibacter* were found to have an effect on AS; and *Faecalibacterium*, *Christensenellaceae R-7* group, *Streptococcus*, *Ruminiclostridium 9*, and *Erysipelatoclostridium* were found to have an effect on NC (Figure 2B). We assumed that these genera reflected the differences in the intestinal microbiomes of the healthy and atopic dermatitis-afflicted individuals and used them as the indicators (or explanatory variables) in the analyses in subsections (Section “3.2. SEM,” “3.3. Comparison of latent variable values between groups in the structural equation model,” “3.4. Estimation of the latent variable values,” “3.5. Atopic dermatitis morbidity probability estimation model with latent variable scores as explanatory variables,” and “3.6. Comparison between groups using the atopic dermatitis morbidity probability estimation model and the latent variable values as explanatory variables”).

### 3.2. SEM

**FIGURE 3:**
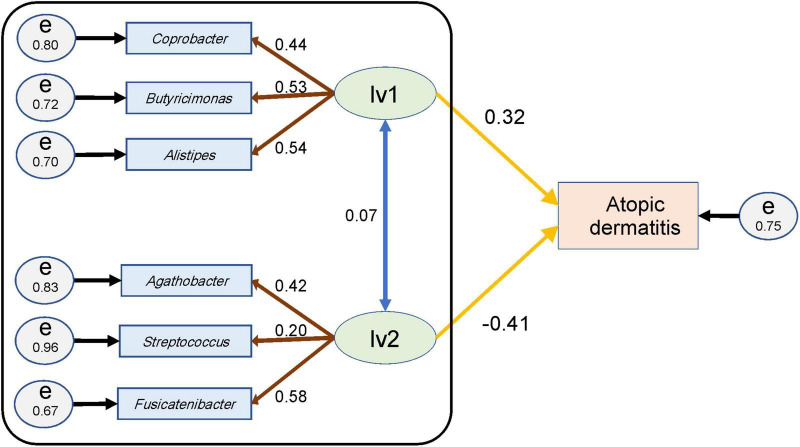
Structural equation model for females and the parameter values. An ellipse (lv1 or lv2) represents a latent variable, and a rectangle (atopic dermatitis or genus name) represents an observed variable or indicators. Circles (e) represent the residual terms for each observed variable or indicator and the numerical value on the circle represents the residual variance. The numerical value of the double-headed blue arrow represents the correlation coefficient between the latent variables; that of the brown arrow represents the loading value from the latent variable to the indicator of the genera; and that of the yellow arrow represents the path coefficient from each latent variable to the observed variable for atopic dermatitis. The goodness-of-fit indices of this structural equation model were goodness-of-fit index (GFI) = 0.95, adjusted GFI (AGFI) = 0.85, and the root mean square error of approximation (RMSEA) = 0.06. The black framed part indicates the SEM portion that was used for estimating latent variable values in subsection (Section “3.4. Estimation of the latent variable values”).

Starting point model structures were constructed first (Supplementary Figures 2, 3), and the resultant model structures obtained after the model modification steps are shown in Figure 3 and Supplementary Figure 4. In females, *Erysipelatoclostridium*, *Oscillibacter*, and *Ruminococcaceae UCG-005* were excluded from the indicators as a result of the model modifications (Figure 3). In males, *Coprobacter*, *Faecalibacterium*, and *Streptococcus* were excluded from the indicators (Supplementary Figure 4).

The data for the NC and AS groups were merged and then used in the structural equation model, for each parameter calculated. In this manner, we investigated how the genera that were set as the indicators explained the variables that represented atopic dermatitis morbidity using the assumed latent variables when focusing on healthy and atopic dermatitis-afflicted individuals.

For women, the standardized path coefficient from latent variable 1 (lv1), which was assumed to have a positive effect on atopic dermatitis, to the atopic dermatitis morbidity variable was 0.32 ± 0.11 (*p* < 0.01); and the standardized path coefficient from latent variable 2 (lv2), which was assumed to have a negative effect on atopic dermatitis, to the atopic dermatitis morbidity variable was −0.41 ± 0.12 (*p* < 0.01). The standardized factor loading values from lv1 to each of indicators of lv1 were as follows: 0.54 ± 0.10 for *Alistipes*, 0.53 ± 0.10 for *Butyricimonas*, and 0.44 ± 0.09 for *Coprobacter*. The standardized factor loading values from lv2 to each indicator of lv2 was as follows: 0.58 ± 0.11 for *Fusicatenibacter*; 0.42 ± 0.09 for *Agathobacter*; and 0.20 ± 0.08 for *Streptococcus*. The standardized factor loading values from the latent variables to each indicator were all significant at *p* < 0.05. The residual variance of the atopic dermatitis morbidity variable was 0.75, and the two assumed latent variables in the structural equation model for females explained approximately 25% of the variance of the atopic dermatitis morbidity variables (Figure 3 and Table 3). The GFI of the structural equation model for females was 0.95, AGFI was 0.85, and RMSEA was 0.06 (RMSEA < 0.08 represents adequate fitting; Browne and Cudeck, 1993). In males, the standardized path coefficient from latent variable lv1 to the atopic dermatitis morbidity variable was 0.96 ± 0.93 (*p* = 0.30), and the standardized path coefficient from latent variable lv2 to the atopic dermatitis morbidity variable was −0.90 ± 0.93 (*p* = 0.33). The standardized factor loading values from lv1 to each indicator of lv1 was 0.30 ± 0.13 (*p* = 0.02) for *Collinsella* and 0.80 ± 0.22 (*p* < 0.01) for *Oscillibacter*. The standardized factor loading values from lv2 for each indicator of lv2 were 0.77 ± 0.24 (*p* < 0.01) for *Ruminiclostridium* 9, −0.20 ± 0.13 (*p* = 0.13) for the *Christensenellaceae R-7* group, and 0.18 ± 0.12 (*p* = 0.12) for *Erysipelatoclostridium*. The residual variance of the atopic dermatitis morbidity variable was 0.42 (Supplementary Figure 4 and Supplementary Table 1). The GFI of the SEM for males was 0.93, AGFI was 0.75, and RMSEA was 0.06. In males, the *p*-values for the parameters from lv1 or lv2 to the atopic dermatitis morbidity variable were ≥ 0.1 for the two latent variable models, and the structural equation model could not be constructed (Supplementary Table 2).

**TABLE 3:** Results of the structural equation modeling (SEM) for women.

lhs^a^	op^b^,^j^	rhs^c^	Stand-ardized parameters^d^	*se* ^e^	*Z*-value^f^	*P*-value^g^	95% confidence limits	
							Lower limit^h^	Upper limit^i^
lv1	= ∼	*Alistipes*	0.54	0.10	5.32	<0.01	0.34	0.75
lv1	= ∼	*Butyricimonas*	0.53	0.10	5.16	<0.01	0.33	0.73
lv1	= ∼	*Coprobacter*	0.44	0.09	4.87	<0.01	0.27	0.62
lv2	= ∼	*Fusicatenibacter*	0.58	0.11	5.14	<0.01	0.36	0.80
lv2	= ∼	*Agathobacter*	0.42	0.09	4.63	<0.01	0.24	0.59
lv2	= ∼	*Streptococcus*	0.20	0.08	2.59	0.01	0.05	0.36
Atopic dermatitis	∼	lv1	0.32	0.11	2.76	0.01	0.09	0.54
Atopic dermatitis	∼	lv2	−0.41	0.12	−3.54	<0.01	−0.64	−0.18
*Alistipes*	∼∼	*Alistipes*	0.70	0.11	6.31	<0.01	0.48	0.92
*Butyricimonas*	∼∼	*Butyricimonas*	0.72	0.11	6.68	<0.01	0.51	0.93
*Coprobacter*	∼∼	*Coprobacter*	0.80	0.08	9.92	<0.01	0.64	0.96
*Fusicatenibacter*	∼∼	*Fusicatenibacter*	0.67	0.13	5.15	<0.01	0.41	0.92
*Agathobacter*	∼∼	*Agathobacter*	0.83	0.07	11.02	<0.01	0.68	0.97
*Streptococcus*	∼∼	*Streptococcus*	0.96	0.03	30.32	<0.01	0.90	1.02
Atopic dermatitis	∼∼	Atopic dermatitis	0.75	0.12	6.50	<0.01	0.52	0.98
lv1	∼∼	lv1	1.00	0.00	NA	NA	1.00	1.00
lv2	∼∼	lv2	1.00	0.00	NA	NA	1.00	1.00
lv1	∼∼	lv2	0.07	0.10	0.69	0.49	−0.13	0.27
*Alistipes*	∼1		0.00	0.09	0.00	1.00	−0.18	0.18
*Butyricimonas*	∼1		0.00	0.08	0.00	1.00	−0.16	0.16
*Coprobacter*	∼1		0.00	0.08	0.00	1.00	−0.16	0.16
*Fusicatenibacter*	∼1		0.00	0.10	0.00	1.00	−0.20	0.20
*Agathobacter*	∼1		0.00	0.07	0.00	1.00	−0.13	0.13
*Streptococcus*	∼1		0.00	0.05	0.00	1.00		0.11
Atopic dermatitis	∼1		0.00	0.00	NA	NA	0.00	0.00
lv1	∼1		0.00	0.00	NA	NA	0.00	0.00
lv2	∼1		0.00	0.00	NA	NA	0.00	0.00

This table shows the results from the SEM shown in Figure 3.

^a^Components on the left-hand side of the SEM.

^b^Operators for each equation in the SEM.

^c^Components on the right-hand side of the SEM.

^d^Standardized estimated value for each parameter in the SEM. If op is = ∼ this column represents standardized factor loading of indicator in rhs, which consists latent variable in lhs. If op is ∼ this column represents the standardized path coefficient from the latent variable in rhs to the observed variable in lhs. If op is ∼∼, this column represents standardized residual variance of the variable in lhs or rhs (lhs = rhs case) or correlation between variables in lhs and rhs (lhs ≠ rhs case). If op is ∼1, this column represents an intercept of the variable in the lhs.

^e^Standard error for the index in the standardized parameters column.

^f^*Z*-values from the statistical test with a null hypothesis for the index when the standardized parameters column is zero.

^g^*P*-values from the statistical test with a null hypothesis for the index when the standardized parameters column is zero.

^h^Lower upper bounds of the 95% confidence interval of the standardized parameters column.

^i^Upper bounds of the 95% confidence interval of the standardized parameters column.

^j^= ∼ Indicates that the variable in the rhs column is an indicator of the latent variable in the lhs column; ∼ indicates that the observed variable in the lhs column is a response variable for the equation which represents the path analysis part of the structural equation model and the latent variable in the rhs column is an explanatory variable of the path analysis. If the variables in the lhs and rhs columns are same, the operator ∼∼ indicates that the value in the standardized parameters column is a variance of residual of the variable in the lhs and rhs. If the variables in the lhs and rhs columns are different, the operator ∼∼ indicates that the value in the standardized parameters column is a correlation between the variables in the rhs and the lhs. The operator ∼1 indicates that the value in the standardized parameters column is an intercept of the variable in the lhs.

### 3.3. Comparison of latent variable values between groups in the structural equation model

**FIGURE 4:**
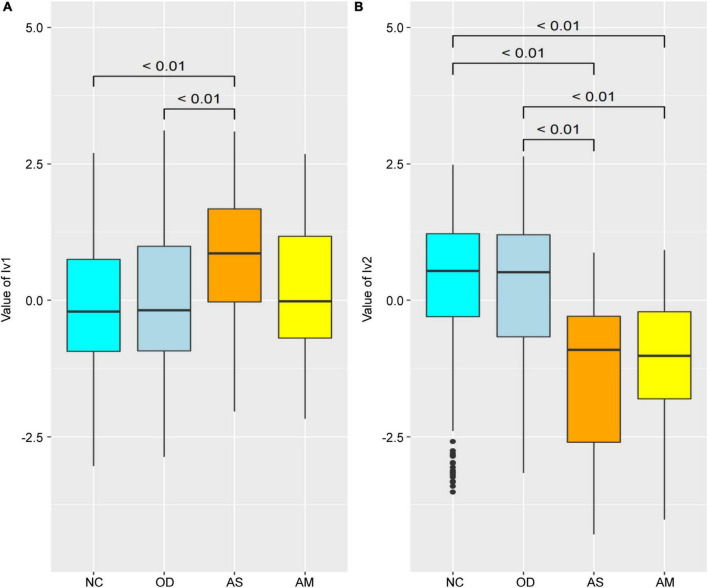
Comparison of the latent variable values between groups for females obtained for each parameter of the structural equation model defined in Figure 3. Latent variable values for **(A)** lv1 and **(B)** lv2. Numerical values on the boxplot represent the *p*-values in the Wilcoxon–Mann–Whitney rank sum test. NC: normal controls (*n* = 321), AS: patients with atopic dermatitis without other diseases (*n* = 45), AM: patients with atopic dermatitis with other diseases (*n* = 75), OD: patients with other diseases but without atopic dermatitis (*n* = 1,669). The bottom line of the box represents the first quartile value, the middle line represents the median, the top line represents the third quartile, and the points represent outliers.

The latent variable values were calculated for each female from each parameter of the SEM in Figure 3 and compared between groups (Figure 4). The lv1 value in females was not significantly different between the NC and OD (*p* = 0.27), while that for the AS was significantly higher than that for the NC (*p* < 0.01). The value for the AM tended to be higher than that for the NC, but there was no significant difference observed (*p* = 0.12). There was no significant difference in the lv2 values for females between the NC and OD groups (*p* = 0.55), and the values for the AS and AM were significantly lower than those of the NC (*p* < 0.01).

### 3.4. Estimation of the latent variable values

**FIGURE 5:**
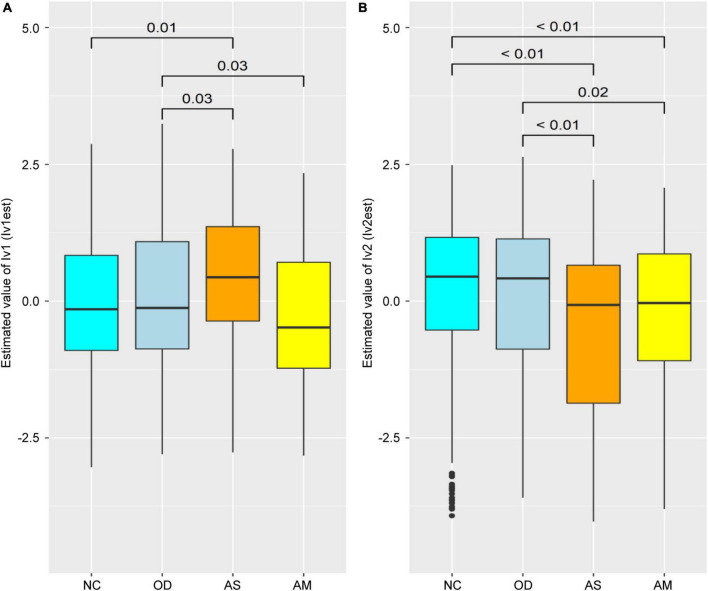
Comparison of the latent variable values between groups for females estimated from the structural equation model in this figure. Estimated latent variable values for **(A)** lv1 and **(B)** lv2. Numerical values on the boxplots represent the *p*-values in the Wilcoxon–Mann–Whitney rank sum test. NC: normal controls (*n* = 321), AS: patients with atopic dermatitis without other diseases (*n* = 45), AM: patients with atopic dermatitis with other diseases (*n* = 75), OD: patients with other diseases but without atopic dermatitis (*n* = 1,669). The bottom line of the box represents the first quartile value, the middle line represents the median, the top line represents the third quartile, and the points represent outliers.

A SEM was constructed to estimate the lv1 and lv2 values for females when the participant’s atopic dermatitis morbidity was unknown (Figure 3 black frame), by extracting the measurement equation portion of the initial SEM in Figure 3. The estimated latent variable values tended to be lower for lv1 and higher for lv2, when compared with the values in Figure 4 for the AS and AM groups. However, the tendency of significant differences in the comparison of the estimated latent variable values between the groups was the same as in Figures 4, 5.

### 3.5. Atopic dermatitis morbidity probability estimation model with latent variable scores as explanatory variables

**FIGURE 6:**
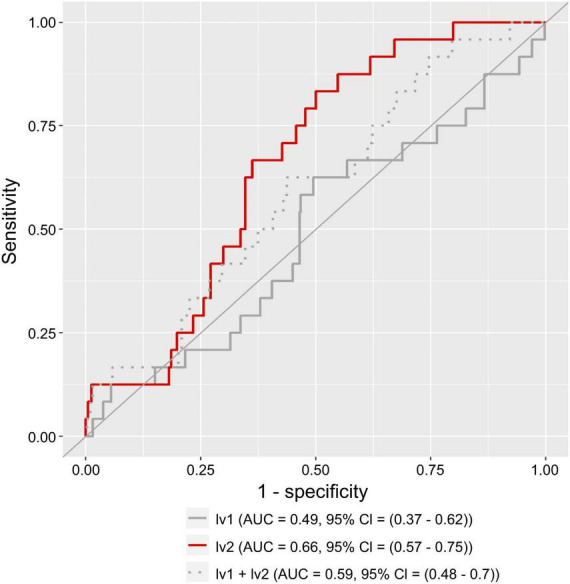
Receiver operating characteristic (ROC) curve for a logistic regression model using lv1 or lv2 as the explanatory variables. The solid gray and red lines represent the results of the model with lv1 and lv2 as the explanatory variables, respectively. The dashed gray line represents the results of the model with both lv1 and lv2 as the explanatory variables. AUC represents the area under the curve, and a 95% CI represents the 95% confidence interval of the AUC.

Figure 6 shows that, for women, the AUC was 0.66 (95% confidence interval (CI): 0.57–0.75) for the model using only lv2 (Figure 6 red line), 0.49 (95% CI: 0.37–0.62) for the model using only lv1 (Figure 6 polygonal gray line), and 0.59 (95% CI: 0.48–0.70) for the model using lv1 and lv2 (Figure 6 dotted gray line). The model using only lv2 had the best accuracy (the highest AUC) of the models assessed.

### 3.6. Comparison between groups using the atopic dermatitis morbidity probability estimation model and the latent variable values as explanatory variables

**FIGURE 7:**
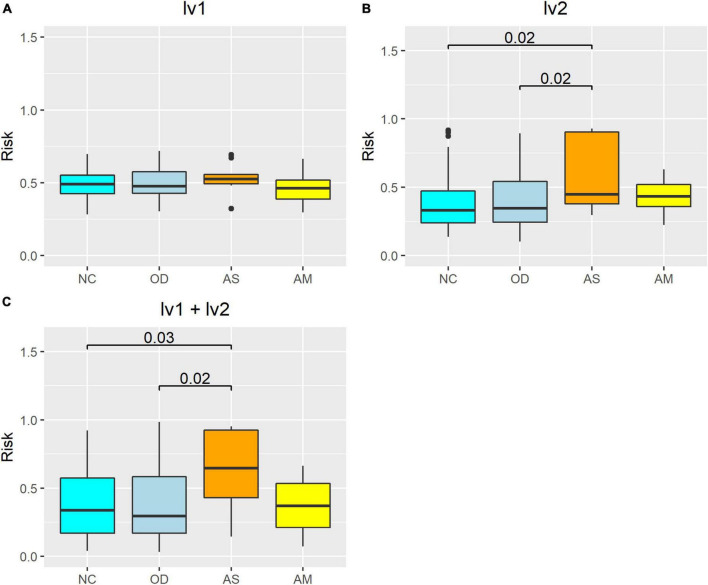
Atopic dermatitis morbidity probability estimation model using the verification population, the latent variables as explanatory variables, and between-group comparisons. The risk on the vertical axis represents the atopic dermatitis morbidity probability calculated from the logistic regression models. The numerical values on the boxplot represent the *p*-values in the Wilcoxon–Mann–Whitney rank sum test. Calculation results from the model with latent variables **(A)** lv1, **(B)** lv2, and **(C)** lv1 and lv2 as the explanatory variables. NC: normal controls (*n* = 321), AS: patients with atopic dermatitis without other diseases (*n* = 45), AM: patients with atopic dermatitis with other diseases (*n* = 75), OD: patients with other diseases but without atopic dermatitis (*n* = 1,669). The bottom line of the box represents the first quartile value, the middle line represents the median, the top line represents the third quartile, and the points represent outliers.

The atopic dermatitis morbidity probabilities were estimated for the verification population using an atopic dermatitis morbidity estimation model with latent variables as explanatory variables, after which the results were compared between groups (Figure 7). When lv1 was used as the only explanatory variable, the estimated atopic dermatitis morbidity probability was not significantly different between the four groups (Figure 7A). When both lv1 and lv2 were used as the explanatory variables, the atopic dermatitis morbidity probability in AS was significantly higher than in NC (*p* < 0.05), but no significant differences were observed between the AM and NC, and no increasing tendency was observed (Figure 7C). Finally, when only lv2 was used as the explanatory variable, the atopic dermatitis morbidity probability of AS was significantly higher than that of NC (*p* < 0.05), and the atopic dermatitis morbidity probability of AM did not show any significant differences from that of the NC, but there was an increasing tendency (Figure 7B).

## 4. Discussion

There are over 1,000 species of intestinal bacteria in the human gut (Rajilić-Stojanović and de Vos, 2014; Yang et al., 2020), and these bacteria may interact each other. Therefore, it is necessary to consider explanatory variables that reflect this interaction when disease risk is estimated using human gut microbiome information. SEM is a useful method that can achieve this because it constructs latent variables consisting of indicators with common characteristics (Dragan and Topolšek, 2014; Andersson and Yang-Wallentin, 2021). Furthermore, the number of latent variables and combination of indicators of latent variables can be set freely; therefore, interpretation of latent variables is easier than principal components. In our SEM, the latent variables in females could be interpreted as follows: We assumed that there are two latent variables, one assumed to have a positive effect on atopic dermatitis (lv1) and the other assumed to have a negative effect on atopic dermatitis (lv2). Each indicator was assigned to latent variables by referring to sign of the indicator’s effect size. We obtained the SEM shown in Figure 3 and Table 3. The genera of bacteria that comprise lv1 are all reportedly involved in inflammatory reactions in the body. For example, *Alistipes* is reported to be involved in inflammatory responses in human and mouse studies (Parker et al., 2020) and is common in patients with chronic obstructive pulmonary disease (Bowerman et al., 2020). *Coprobacter* is also reported to be increased in patients with autism spectrum disorder and constipation, with a concomitant decrease in butyrate-producing bacteria (Liu et al., 2019). Butyrate inhibits histone deacetylase and suppresses intestinal inflammation-induced macrophage function (Chang et al., 2014). Furthermore, *Butyricimonas* is reportedly involved in the production of inflammatory cytokines such as interleukin-1β and transforming growth factor-β1 (Kim et al., 2019). Thus, lv1 could show the association between the intestinal microbiota and atopic dermatitis as an enhanced inflammatory response involving *Alistipes*, *Coprobacter*, and *Butyricimonas*. However, one of the genera in lv2 is *Agathobacter*, which is a butyrate-producing bacterium (Rosero et al., 2016) that is reportedly reduced in patients with non-febrile *Mycoplasma pneumoniae* pneumonia when compared with healthy children (Jiang et al., 2022). *Fusicatenibacter* is also a butyrate-producing bacterium; *Fusicatenibacter saccharivorans* is decreased in patients with ulcerative colitis, and its administration is reported to improve enteritis in mice (Takeshita et al., 2016). Furthermore, *Streptococcus salivarius* is shown to have anti-inflammatory effects (Kaci et al., 2014), and the abundance of *Streptococcus* is reported to be lower in patients with transient atopic dermatitis than in healthy individuals (Park et al., 2020). Thus, since the bacterial genera in lv2 are all related to the suppression of inflammatory responses, lv2 is considered a latent variable related to the suppression of inflammatory responses. In other words, lv2 could show the relationship between the intestinal microbiome and atopic dermatitis as an inhibitory effect on inflammatory reactions involving *Agathobacter*, *Fusicatenibacter*, and *Streptococcus* that comprise lv2. As mentioned above, SEM provides easily interpretable variables that reflect the interaction of the indicators.

Structural equation modeling contains path analysis (Dragan and Topolšek, 2014; Andersson and Yang-Wallentin, 2021); therefore, the influence of latent variables on the objective variable can be estimated as path coefficients. In women, the path coefficient from lv1 to the atopic dermatitis incidence status showed a variance of 0.32 ± 0.11 (*p* < 0.01), and the path coefficient from lv2 to the atopic dermatitis incidence status showed a variance of −0.41 ± 0.12 (*p* < 0.01; Figure 3) in our SEM. The GFI of the SEM was 0.95, AGFI was 0.85, and RMSEA was 0.06 (RMSEA < 0.08 represents adequate fitting; Browne and Cudeck, 1993). Recall a flexibility of model construction of SEM, it is possible to find more effective latent variables by adding or removing indicators to the model referring these indices. Actually, we obtained lv1 and lv2 in Figure 3 by such way.

In men, a SEM could not be constructed. A parallel analysis using the genera in Figure 2B as variables showed that the estimated number of latent factors was 0 (Supplementary Figure 5), indicating that these genera showed high uniqueness values and do not constitute latent variables.

As discussed above, SEM can provide variables which reflect the interaction of bacteria in human gut microbiome and their design method. Especially, a flexibility of model construction of SEM allows more complex variable design than previous methods, for example, the stepwise variable selection in multivariate regression analysis. These properties of SEM can enhance construction of models which try to estimate a disease risk from human gut microbiome information.

We constructed models estimate atopic dermatitis risk using latent variables in our SEM in Figure 3. Assuming actual clinical settings, the model constructions and accuracy assessments of the models were performed using the NC (healthy participants), AS (patients affected with only with atopic dermatitis), AM (patients affected with atopic dermatitis and other diseases), and OD (patients affected with diseases other than atopic dermatitis) groups.

First, we compared values of the latent variables between the groups. The values for lv1 were significantly higher for the AS group than the NC and OD groups (Figure 4A). On the other hand, there were no significant increases in the AM lv1 values with NC and OD. The values for lv2 were significantly lower in the atopic dermatitis-affected groups (AS and AM) than in the atopic dermatitis -unaffected groups (NC and OD; Figure 4B). These results imply that lv1 is influenced by fluctuations in the gut microbiota associated with other diseases and lv2 could be a latent variable that captures the association with atopic dermatitis with some reliability, regardless of whether the patient has a disease other than atopic dermatitis, that is, without being affected by the variations in the gut microbiota associated with other diseases.

Next, a SEM was then constructed to estimate the values of lv1 and lv2 when the participant’s atopic dermatitis status was unknown (Figure 3 black frame). In general, the value of each latent variable in a SEM is calculated using all observed variables and all indicators in the model (Dragan and Topolšek, 2014; Andersson and Yang-Wallentin, 2021). However, when using gut microbiota analysis data to estimate the risk of disease, it is necessary to estimate latent variables in the SEM with the observed variables if disease prevalence is unknown. Therefore, we constructed a SEM (Figure 3 black frame) by extracting the measurement equation part from the original SEM (Figure 3) and used this to estimate the values of the latent variables lv1 and lv2. The resulting latent variable value estimates (lv1est and lv2est) showed a similar trend to the latent variable values calculated by the original SEM, and the lv2est was significantly lower in the atopic dermatitis-affected group (AS, AM) than in the atopic dermatitis-unaffected group (NC,OD; Figure 5). This suggests that lv2 could be used for women when constructing a model to estimate the probability of atopic dermatitis (disease risk) from the gut microbial community composition information.

We constructed a disease risk estimation model for atopic dermatitis using a logistic regression model, in which lv2 are explanatory variables for the atopic dermatitis incidence status variable. The model is designed to estimate the disease risk for atopic dermatitis in new participants using the following procedure: (1) A model is trained using each participant’s lv2 (explanatory variables) calculated from the intestinal bacteria analysis data and atopic dermatitis prevalence variables for each participant collected in advance, and a model is constructed to estimate the disease risk for atopic dermatitis from the lv2; (2) For new participants, lv2est are calculated and the values are fit to the model in (1) to estimate disease risk. For comparison, we also constructed a model with lv1 as the explanatory variable and both lv1 and lv2 as explanatory variables. Estimates of disease risk were calculated by fitting the lv1 and/or lv2 estimates (lv1est and/or lv2est) of the validation population to the three models constructed using data from this training population, and the accuracy of each model was compared using ROC analysis. The results showed that the model with lv2 as the explanatory variable had the highest accuracy (AUC = 0.66; satisfactory; Trifonova et al., 2014) and the model with lv1 as the explanatory variable had the lowest accuracy (AUC = 0.49; unsatisfactory; Trifonova et al., 2014). The accuracy of the model with both lv1 and lv2 as explanatory variables was lower (AUC = 0.59; unsatisfactory; Trifonova et al., 2014) than the model with lv2 alone (Figure 6). The results of the intergroup comparisons for the calculated estimates for disease risk for atopic dermatitis also show a similar trend (Figure 7). These results may reflect the results of Figures 4, 5, in which lv1 may be affected by fluctuations in the intestinal microbiota associated with diseases other than atopic dermatitis, making it difficult to capture the association with atopic dermatitis, while lv2 may be unaffected by fluctuations in the intestinal microbiota associated with other diseases and may be somewhat stable and capture the association with atopic dermatitis, and these results are reflected in Figures 4, 5.

To compare these results with the disease risk estimates for atopic dermatitis made using individual bacterial genera, we compared the accuracy of the disease risk estimation model for atopic dermatitis with the relative abundance of individual bacterial genera as explanatory variables and further assessed the results using AUC analysis. The accuracy of the model constructed in this study using lv2 as the explanatory variable was higher than that of the model using the CLR-transformed abundance of individual genera as the explanatory variable, except for the model using *Agathobacter* as the explanatory variable (Supplementary Table 1). The model with only *Agathobacter* as the explanatory variable showed higher AUC values than the model using lv2, but it is limited as it requires that the association between the gut microbiota and atopic dermatitis is captured by the amount of *Agathobacter* alone. For example, lv2, which in this study consisted of three genera of bacteria (indicators), was suggested to be a latent variable related to the inhibitory effects of inflammatory responses, but it is possible that there are participants whose *Agathobacter* abundance is not greatly different from that of healthy participants, and that the decrease in lv2 is caused by a decrease in the relative abundance of *Fusicatenibacter* or *Streptococcus* (in Figure 3, factor loadings from lv2 are all positive, and therefore, the decrease in lv2 is considered a decrease in the abundance of *Fusicatenibacter* or *Streptococcus* when there is no change in the abundance of *Agathobacter*). For such participants, a model that uses only the amount of *Agathobacter* present as an explanatory variable cannot accurately estimate the participant’s risk for disease.

In this study, we showed it is possible to use latent variables in SEM as explanatory variables to estimate the disease risk with satisfactory accuracy (Figure 6 and Supplementary Table 1). We also noted the following properties of SEM: (1) latent variables in SEM are thought to reflect, to some extent, the interactions of multiple indicators, (2) a high flexibility of model construction makes more complex variable design possible compared with previous methods. Therefore, we showed SEM can be a novel choice for construction of explanatory variables to estimate disease risk. In particular, the high flexibility of model construction in SEM allows identification of latent variables that have better accuracy with model modifications. In future research, SEM using other microbiome data (e.g., gene expression data) or metadata of participants (e.g., nutritional intake data) may be constructed, and the whole picture of the relationship between microbial community composition and disease may be described as SEM with these variables. Such SEM will advance the construction of models to estimate the disease risk, and these risk models will support diagnosis, treatment, and prevention of diseases in clinical settings.

## Data availability statement

The 16S rRNA amplicon sequencing data presented in the study are deposited in the European Nucleotide Archive (ENA) repository (https://www.ebi.ac.uk/ena/browser/home), accession number: PRJEB57381. The other raw data supporting the conclusions of this article will be made available by the authors, without undue reservation.

## Ethics statement

The studies involving human participants were reviewed and approved by the Research Ethics Committee of RIKEN (approval number: Wako 3 27-22). The patients/participants provided their written informed consent to participate in this study.

## Author contributions

HT, HM, and YB contributed to the conception and design of the study. YB conducted the DNA extraction, 16S rRNA amplicon sequencing, and questionnaire survey. HT, TI, and JK performed data curation and performed statistical and data analysis. HT, KO, KH, and HM wrote specific sections in the first draft of the manuscript. All authors contributed to manuscript revision, read, and approved the submitted version.

## Footnotes

1
https://www.claident.org/

2
https://benjjneb.github.io/dada2/tutorial.html

3
https://benjjneb.github.io/dada2/bigdata.html
