# Supplementary material for: Method for estimating disease risk from microbiome data using structural equation modeling
Source: Front Microbiol. 2023 Jan 26;14:1035002. doi: 10.3389/fmicb.2023.1035002 (PMC9909428; doi:10.3389/fmicb.2023.1035002)
Supplement: Supplementary file 1 [file Data_Sheet_1.docx]

Supplementary Material

# Supplementary Figures and Tables

## Supplementary Figures





**Supplementary Figure 1.** **Results of the Monte Carlo sampling which were processed using ALDEx2 software.** Effect size was calculated 500 times and arranged in descending order based on the absolute value. In this way, 500 lists were obtained identifying the top 20 genera in terms of effect size and the genera that intersected across these lists were then identified, and this was conducted for both the (**A)** female and (**B)** male data. The x-axis represents the number of trials conducted to calculate the different effect sizes, and the vertical axis represents the number of genera that continue to be in the top 20.


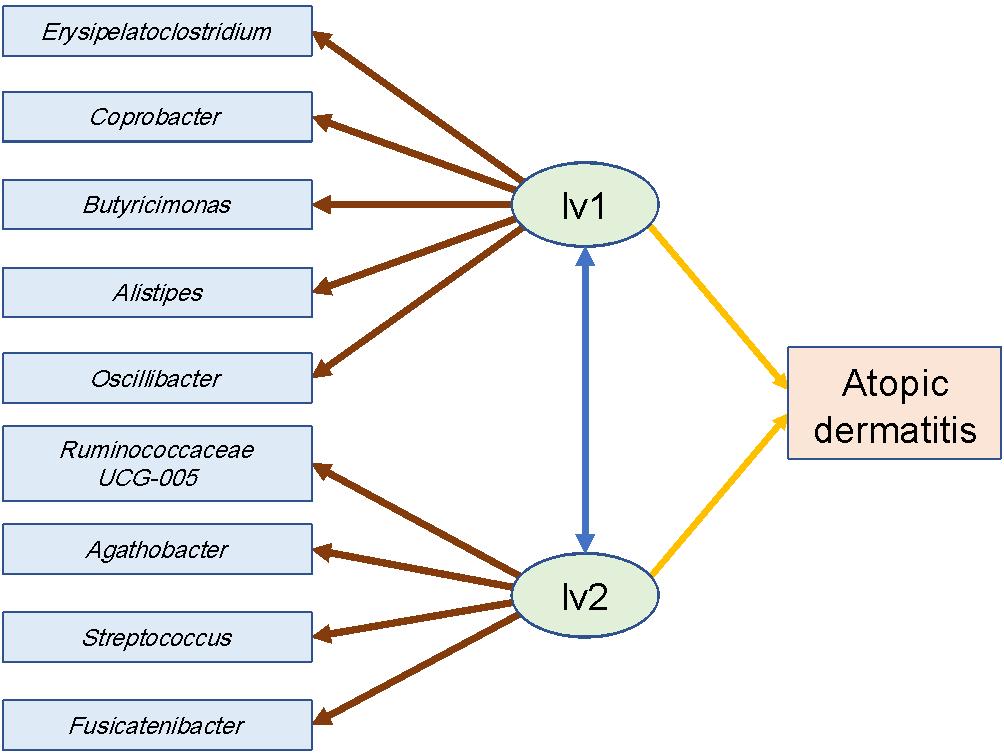


**Supplementary Figure 2.** **First hypothesized structural equation model for women.** Ellipses (lv1 or lv2) represent latent variables, and rectangles (atopic dermatitis or genus name) represent observed variables or indicators. After several model modification steps, the structural equation model in Figure 3 was obtained from this model.


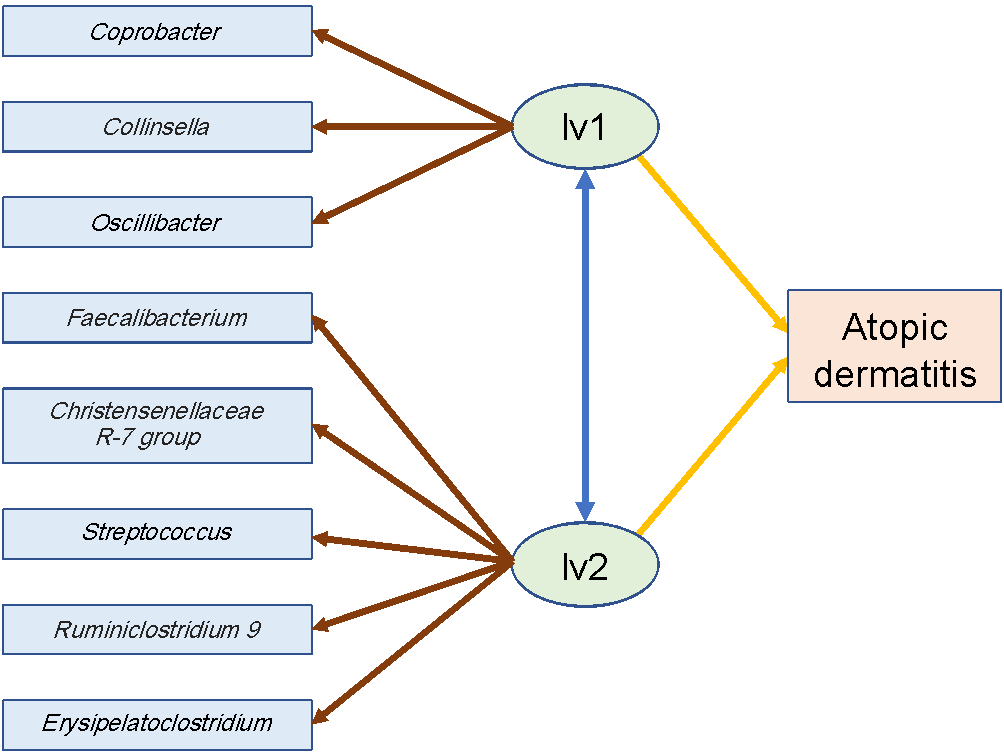


**Supplementary Figure 3.** **First hypothesized structural equation model for men.** Ellipses (lv1 or lv2) represent latent variables, and rectangles (atopic dermatitis or genus name) represent observed variables or indicators. After several model modification steps, the structural equation model in Figure S4 was obtained from this model.


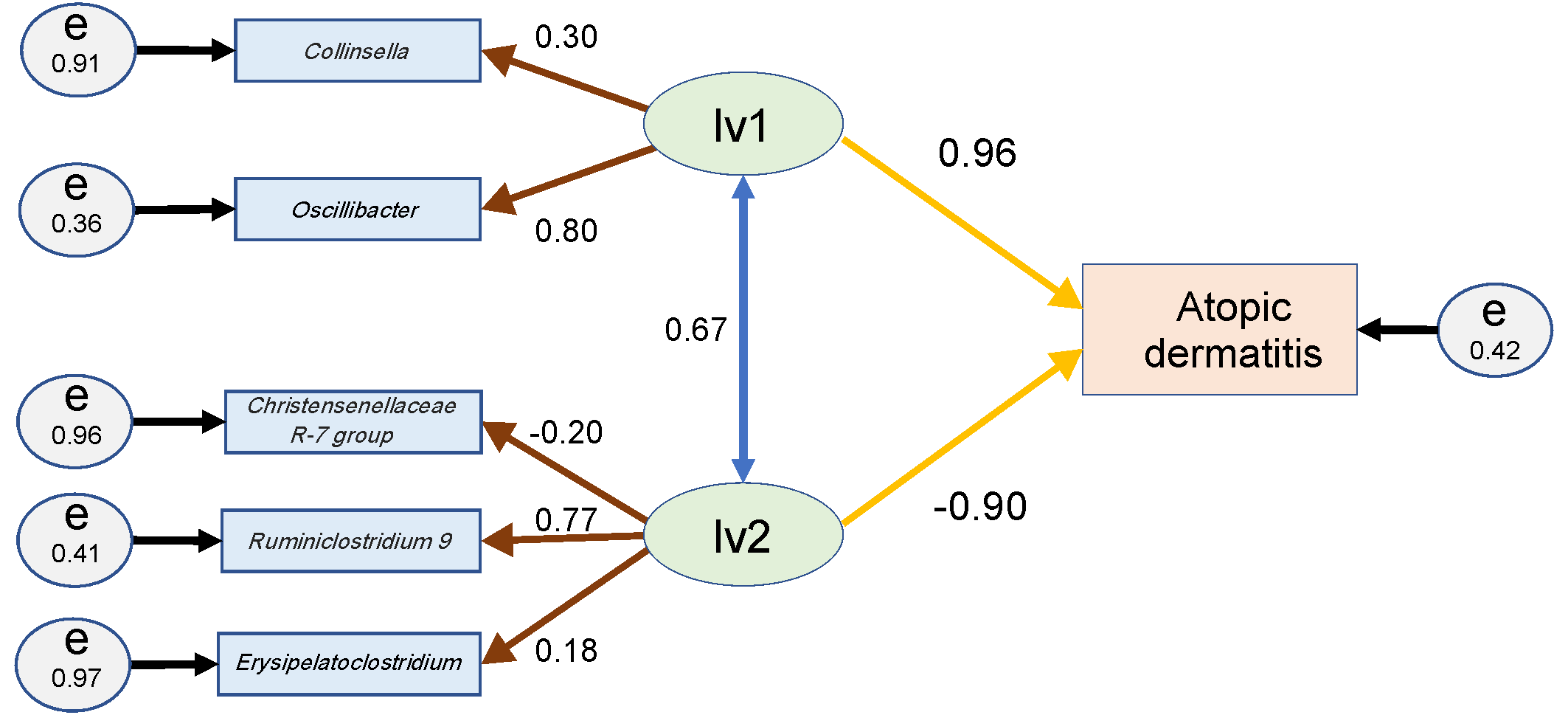


**Supplementary Figure 4.** **Final structural equation model for men.** Ellipses (lv1 or lv2) represent latent variables and rectangles (atopic dermatitis or genus name) represent observed variables or indicators. Circles (e) represent the residual terms for each observed variable or indicator and the numerical value on each circle represents the residual variance. The numerical value alongside the double-headed blue arrow represents the correlation coefficient between the latent variables; those alongside the brown arrows represent the loading values from the latent variables to the indicators of the genera; and those alongside the yellow arrows represent the path coefficients from each latent variable to the observed variable for atopic dermatitis. The goodness-of-fit indices of this structural equation model were Goodness of Fit Index (GFI) = 0.93, Adjusted Goodness of Fit Index (AGFI) = 0.75, and the root mean square error of approximation (RMSEA) = 0.06.


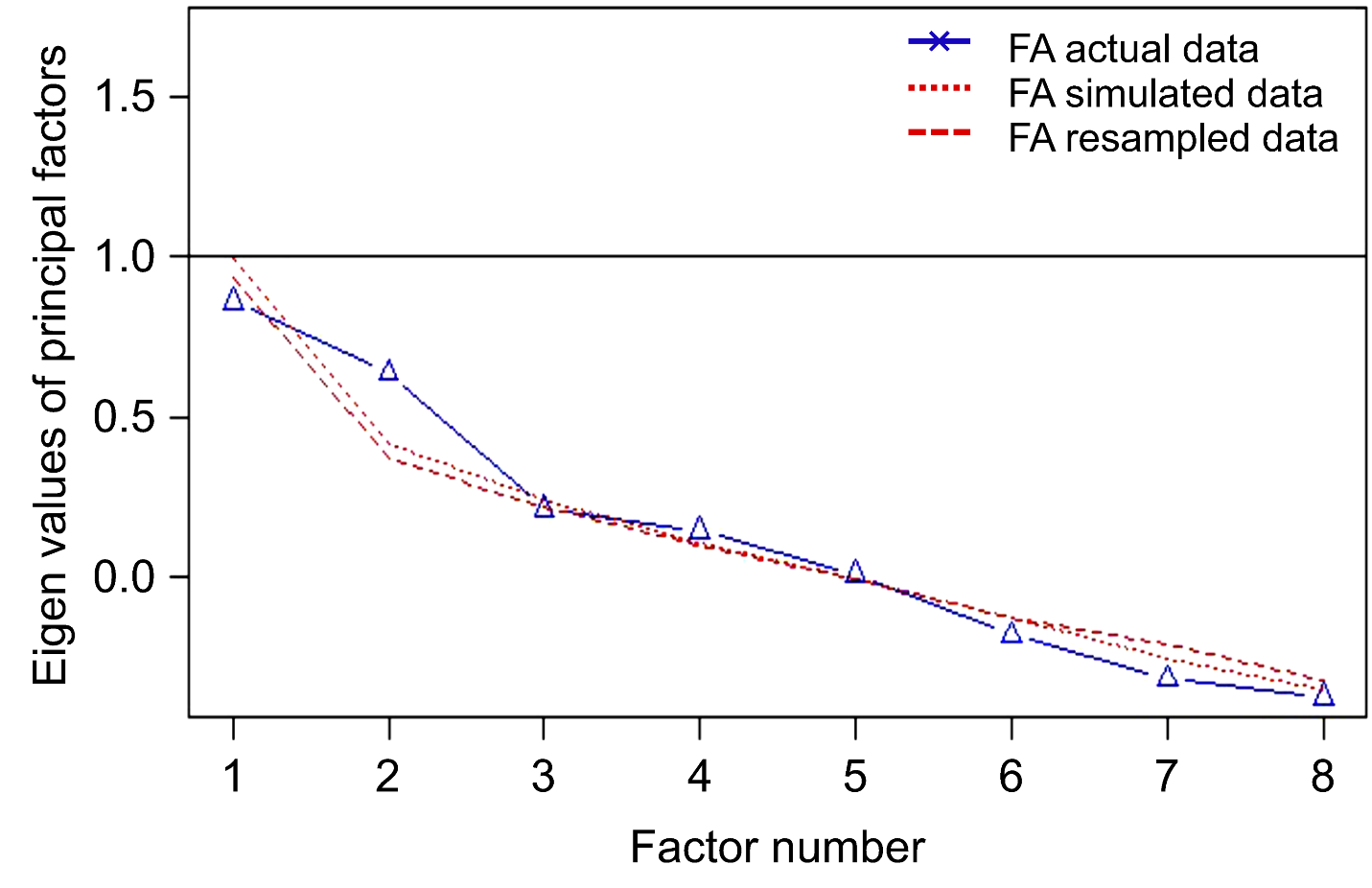


**Supplementary Figure 5.** **Parallel analysis results for the selected genus variables in males.** The fa.parallel function in psych-2.1.9 (Revelle, W. (2021) psych: Procedures for Personality and Psychological Research, Northwestern University, Evanston, Illinois, USA, URL https://CRAN.R-project.org/package=psych Version = 2.1.9,.) was used to conduct a parallel analysis. The rotation of the factor was set to diagonal using rotate = “Promax” and the GPArotation-2014.11-1 (Bernaards, Coen A. and Jennrich, Robert I. (2005) Gradient Projection Algorithms and Software for ArbitraryRotation Criteria in Factor Analysis, Educational and Psychological Measurement: 65, 676-696. URL http://www.stat.ucla.edu/research/gpa) package. The analysis was conducted using generalized weighted least squares (GLS), setting the argument to fm = “gls”. The line for ‘FA Actual Data’ represents the eigen values of the factors derived from the actual CLR transformed abundance data (i.e. eigen values from the correlation matrix for abundance data) for the genera identified in Fig. 2B, if the number of factors is set as an x axis value in factor analysis (FA). Furthermore, the line ‘FA Simulated Data’ represents the eigen values for factors derived from random numbers and the line “FA Resampled Data” represents the eigen values for factors derived from random sampled data taken from the actual data, if the number of factors is set as the x axis value in FA. The blue line does not cross the constant line (Eigen values of factors = 1.0), and this suggests that no factor is derived from the indicators (the genera variables in Figure 2B).

## Supplementary Tables

**Supplementary Table 1.** **Accuracy of the atopic dermatitis morbidity probability estimation model using each genus identified in women as an explanatory variable.**

| Explanatory variable | AUC^1^ | 95% CI^2^ |
| --- | --- | --- |
| *Agathobacter* | 0.68 | 0.56–0.81 |
| *Alistipes* | 0.57 | 0.45–0.69 |
| *Butyricimonas* | 0.49 | 0.37–0.60 |
| *Coprobacter* | 0.49 | 0.35–0.62 |
| *Fusicatenibacter* | 0.55 | 0.43–0.66 |
| *Streptococcus* | 0.62 | 0.48–0.75 |

^1^ area under the curve (AUC) determined using ROC analysis

^2^ 95% confidence interval of the AUC

**Supplementary Table 2.** **Results of the structural equation modeling for Fig. S4.**

| lhs^a^ | op^b,j^ | rhs^c^ | Standardized parameters^d^ | Se^e^ | z value^f^ | p value^g^ | 95% confidence limits | |
| --- | --- | --- | --- | --- | --- | --- | --- | --- |
|  |  |  |  |  |  |  | Lower limit^h^ | Upper limit^i^ |
| lv1 | =~ | *Collinsella* | 0.30 | 0.13 | 2.37 | 0.02 | 0.05 | 0.55 |
| lv1 | =~ | *Oscillibacter* | 0.80 | 0.22 | 3.57 | 0.00 | 0.36 | 1.24 |
| lv2 | =~ | *Ruminiclostridium_9* | 0.77 | 0.24 | 3.17 | 0.00 | 0.29 | 1.24 |
| lv2 | =~ | *Christensenellaceae_R_7_group* | -0.20 | 0.13 | -1.53 | 0.13 | -0.46 | 0.06 |
| lv2 | =~ | *Erysipelatoclostridium* | 0.18 | 0.12 | 1.55 | 0.12 | -0.05 | 0.41 |
| atopic_flg | ~ | lv1 | 0.96 | 0.93 | 1.04 | 0.30 | -0.86 | 2.78 |
| atopic_flg | ~ | lv2 | -0.90 | 0.93 | -0.97 | 0.33 | -2.73 | 0.93 |
| *Collinsella* | ~~ | *Collinsella* | 0.91 | 0.08 | 12.09 | 0.00 | 0.76 | 1.06 |
| *Oscillibacter* | ~~ | *Oscillibacter* | 0.36 | 0.36 | 0.99 | 0.32 | -0.35 | 1.06 |
| *Ruminiclostridium_9* | ~~ | *Ruminiclostridium_9* | 0.41 | 0.37 | 1.09 | 0.27 | -0.32 | 1.14 |
| *Christensenellaceae_R_7_group* | ~~ | *Christensenellaceae_R_7_group* | 0.96 | 0.05 | 18.31 | 0.00 | 0.86 | 1.06 |
| *Erysipelatoclostridium* | ~~ | *Erysipelatoclostridium* | 0.97 | 0.04 | 23.39 | 0.00 | 0.89 | 1.05 |
| atopic_flg | ~~ | atopic_flg | 0.42 | 0.78 | 0.53 | 0.59 | -1.11 | 1.94 |
| lv1 | ~~ | lv1 | 1.00 | 0.00 | NA | NA | 1.00 | 1.00 |
| lv2 | ~~ | lv2 | 1.00 | 0.00 | NA | NA | 1.00 | 1.00 |
| lv1 | ~~ | lv2 | 0.67 | 0.26 | 2.55 | 0.01 | 0.15 | 1.18 |
| *Collinsella* | ~1 |  | 0.00 | 0.11 | 0.00 | 1.00 | -0.22 | 0.22 |
| *Oscillibacter* | ~1 |  | 0.00 | 0.10 | 0.00 | 1.00 | -0.20 | 0.20 |
| *Ruminiclostridium_9* | ~1 |  | 0.00 | 0.20 | 0.00 | 1.00 | -0.39 | 0.39 |
| *Christensenellaceae_R_7_group* | ~1 |  | 0.00 | 0.19 | 0.00 | 1.00 | -0.36 | 0.36 |
| *Erysipelatoclostridium* | ~1 |  | 0.00 | 0.11 | 0.00 | 1.00 | -0.21 | 0.21 |
| atopic_flg | ~1 |  | 0.00 | 0.00 | NA | NA | 0.00 | 0.00 |
| lv1 | ~1 |  | 0.00 | 0.00 | NA | NA | 0.00 | 0.00 |
| lv2 | ~1 |  | 0.00 | 0.00 | NA | NA |  | 0.00 |

This table shows the results from the SEM shown in Figure S4.

^a^ components on the left-hand side of the SEM

^b^ operators for each equation in the SEM

^c^ components on the right-hand side of the SEM

^d^ standardized estimated value for each parameters in the SEM. If op is =~ this column represents standardized factor loading of indicator in rhs which consists latent variable in lhs. If op is ~ this column represents standardized path coefficient from latent variable in rhs to observed variable in lhs. If op is ~~ this column represents standardized residial variance of variable in lhs or rhs (lhs = rhs case) or correlation between variables in lhs and rhs (lhs ≠ rhs case). If op is ~1 this column represents an intercept of the variable in the lhs.

^e^ standard error for the index in the standardized parameters column.

^f^  z-values from the statistical test with a null hypothesis for the index when the standardized parameters column is zero

^g^  p-values from the statistical test with a null hypothesis for the index when the standardized parameters column is zero

^h^ lower upper bounds of the 95% confidence interval of the standardized parameters column

^i^ upper bounds of the 95% confidence interval of the standardized parameters column

^j^ =~ indicates that the variable in the rhs column is an indicator of the latent variable in the lhs column; ~ indicates that the observed variable in the lhs column is a response variable for the equation which represents the path analysis part of the structural equation model and the latent variable in the rhs column is an explanatory variable of the path analysis. If the variables in the lhs and rhs columns are same, the operator ~~ indicates that the value in the standardized parameters column is a variance of resudial of the variable in the lhs and rhs. If the variables in the lhs and rhs columns are different, the operator ~~ indicates that the value in the standardized parameters column is a correlation between the variables in the rhs and the lhs. The operator ~1 indicates that the value in the the standardized parameters column is an intercept of the variable in the lhs.
